# Active Prevalence of *Fusarium falciforme* and *F. acutatum* Causing Basal Rot of Onion in Maharashtra, India

**DOI:** 10.3390/jof10060413

**Published:** 2024-06-07

**Authors:** Ram Dutta, Krishnappa Jayalakshmi, Auji Radhakrishna, Satish Kumar, Vijay Mahajan

**Affiliations:** ICAR-Directorate of Onion and Garlic Research, Pune 410505, Maharashtra, India; radhakrishna17@gmail.com (A.R.); satish.kumar10@icar.gov.in (S.K.); vijay.mahajan@icar.gov.in (V.M.)

**Keywords:** basal rot, *Fusarium falciforme*, *Fusarium acutatum*, onion, disease prevalence

## Abstract

Over the past decade, there have been accumulating reports from researchers, farmers, and field extension personnel on the increasing incidence and spread of onion basal rot in India. Onion basal rot disease is mainly caused by *Fusarium* spp. This study aimed to validate the information on the active prevalence of *F. falciforme* and *F. acutatum* causing *Fusarium* basal rot (FBR) in Maharashtra. A survey was conducted, and the infected plants/bulbs were collected from fields of 38 locations comprising five districts of Maharashtra, namely, Nashik, Aurangabad, Solapur, Ahmednagar, and Pune, in 2023. This disease was prevalent in high-moisture and high-oil-temperature conditions and the symptoms were observed in most of the fields, with the FBR incidence ranging from 17 to 41%. The available data of basal rot incidence from 1998 to 2022 were analyzed, based on which the prevalence of FBR was 11–50%. Tissue from the infected samples of onion bulbs was used for the isolation. The identification was performed based on colony morphology and microscopic features and confirmed through molecular markers using ITS and *Tef*-1α gene primers. Of the ten *Fusarium* isolates collected from selected locations, six species were confirmed as *F. acutatum* and four as *F. falciforme*. The pathogenicity tests performed with onion seedlings and bulbs under moist conditions proved that both *F. acutatum* and *F. falciforme* independently could cause basal rot disease symptoms but with different degrees of virulence. Koch’s postulates were confirmed by reisolating the same pathogens from the infected plants. Thus, the active prevalence of FBR was confirmed in Maharashtra and also, to the best of our knowledge, this is the first report of *F. falciforme* and *F. acutatum* causing basal rot of onion independently in Maharashtra, India.

## 1. Introduction

Onion (*Allium cepa* L.), a fresh spice/vegetable, is consumed all over the world and India is globally the largest producer of onion with an overall production of 31.68 Mt [1]. Though onion is primarily grown for food, it is also used as a traditional medicine in many Asian countries. Averaged over the quinquennial period of 2017 to 2022, India ranked first in area (1.37 million ha) and production (24.25 Mt) of onion in the world. However, the onion productivity (16.40 t/ha) of India ranked at the 90th position, being far lower than many other countries [1].

*Fusarium* is one of the most important genera of fungi due to its wide geographical distribution and morphological and genetic diversity, inciting a variety of plant diseases. *Fusarium* basal rot (FBR) is a seed- and soil-borne disease of *Allium* crops caused by a complex set of *Fusarium* species, viz., *F. oxysporum*, *F. proliferatum*, *F. acutatum*, and *F. falciforme*, in pre- as well as post-harvest conditions.

FBR is an economically significant disease of onion that causes considerable yield losses of up to 50% in the field and 30–40% during the post-harvest storage of bulbs [2]. FBR generally occurs at all stages of *Allium* development [3]; however, seedlings and dormant or post-harvest bulbs are the most susceptible stages. Infected seeds and soil are the source of dispersal. This disease is widespread, particularly in the rainy season or in high-moisture conditions. The damage from basal rot is mostly recorded during storage [4]. Primary infection by *Fusarium* spp. occurs when the fungus penetrates directly into roots or through wounds in roots or the basal parts of bulb scales [5,6,7]. In the early stages of germination, the infection results in the delay of seedling emergence or a damping-off of the seedlings [8,9,10,11]. Under field conditions, FBR-affected plants show symptoms of chlorosis, twisting, wilting, necrosis, bulb discoloration, and rot in the basal parts of the bulb and roots which become more noticeable during the plant’s maturity [12,13]. The disease outcome is the result of a host–pathogen interaction under the influence of environmental conditions. The diversity of *Fusarium* and *Allium* species and the occurrence of mixed infections enhance a multiplicity of FBR disease outcomes [14,15,16].

In India, the incidence of basal rot was first reported in Rajasthan by Mathur and Shukla [17], then in Coimbatore, Tamil Nadu [18]. This disease is more prevalent in the southern region [19,20] of India. In India, since 1963, FBR has been known to be caused by *Fusarium oxysporum* f. sp. *cepae* [21], and in 2014, Ravi et al. [22] reported the association of *F. proliferatum* in basal rot in Andhra Pradesh [23] and then *F. falciforme* [13] and *F. acutatum* [24] causing FBR in Karnataka.

The Indian Council of Agricultural Research-Directorate of Onion and Garlic Research (ICAR-DOGR), the then National Research Centre for Onion and Garlic (NRCOG), Pune, Maharashtra, initiated sporadic surveys from 1998 to record the status of basal rot in Maharashtra, and the disease was recorded with variable intensity ranging from 11 to 50% [25,26,27]. In Maharashtra, basal rot was reported to be majorly caused by *Fusarium oxysporum* f. sp. *cepae* until 2023 [28,29,30,31].

While going through the reports of *F. falciforme* and *F. acutatum* [13,24] causing basal rot (FBR) in Karnataka, we analyzed our samples collected from ten locations covering five districts, namely, Ahmednagar, Aurangabad, Nashik, Pune, and Solapur of Maharashtra, in 2023 and recorded that the basal rot incidence was caused by *F. falciforme* and *F. acutatum*. This indicates that the active prevalence of FBR in India is increasing day by day, which may become a major disease in years to come, making it an important issue for onion growers. The present study reports the prevalence and expansion of the FBR disease incidence caused by *F. falciforme* and *F. acutatum* in onion-growing regions of Maharashtra, India.

## 2. Materials and Methods

### 2.1. Survey and Sample Collection

ICAR-Directorate of Onion and Garlic Research, Pune, is the nodal agency in India with a mandate to plan, monitor, and evaluate the research on onion and garlic through independent and coordinated activities through its All-India Network Research Program on Onion and Garlic (AINRPOG), which currently has 28 centers located in different agroclimatic zones -. The PAN-India multilocational trials for improvement, production, and protection activities have been conducted since the inception of the institute. The various sorts of multidimensional data, including prevalent diseases of onion and disease incidence and severity, are collected in various research trials and the data so produced are documented every year in the Annual Report of AINPROG. The data on disease surveys of onion-growing areas of Maharashtra from 1998 to 2022 were collected from available reports [25,26,27], except for 2001 to 2006. Furthermore, a fresh survey was conducted in five different districts of Maharashtra, viz., Ahmednagar, Aurangabad, Nashik, Pune, and Solapur. A total of 38 villages from 11 blocks were surveyed in 2023 to understand the prevalence of basal rot. During the survey, samples from infected bulbs/plants were also collected from selective locations for the isolation and identification of pathogens. The percent disease incidence from each location was calculated using the following formula [32]:

$$Disease\;incidence\;\left(\%\right)=\frac{Number\;of\;infected\;plants}{Total\;number\;of\;observed\;plants}\times 100$$

### 2.2. Isolation and Identification of Isolates from Basal Rot Bulbs/Plants

Plants and bulbs showing symptoms of basal rot were collected from the surveyed locations, transported to the laboratory in sterile autoclaved bags, and processed for pathogen isolation using the method described by Rangaswamy, 1958 [33], and the subsequent cultural, morphological, and molecular characterization was performed using standard protocols. Briefly, the affected basal plate/bulb tissues were cut into small segments (approximately 5 mm long) and each piece was surface-sterilized by dipping in 1% sodium hypochlorite for 1 min and then washing thrice in sterile distilled water. Then, the infected tissues were placed on potato dextrose agar (PDA) in Petri plates and incubated at 25 ± 2 °C for 7 days. Single hyphal tips were transferred onto fresh PDA plates in such a way as to obtain a pure culture of isolates. The purification of cultures was performed by taking mycelial plugs (5 mm) from seven-day-old actively growing mycelia and placing them in 2% water agar plates. The plates were incubated for 48 h, and then observed under a compound microscope (Primostar, Carl Zeiss, Oberkochen, Germany) to locate the hyphal tip using the low-power objective. The hyphal tips were placed on PDA plates and slants to maintain as pure cultures. The pure cultures were stored at 4 °C for further studies. Thirty-eight total isolates were obtained from distinct samples. Among the 38 samples, 10 selective fungal isolates, named OGRDFW1 to OGRDFW10, from different districts were subjected to cultural, morphological, and molecular identification. The cultural and morphological characterization was performed using 7-day-old culture with a compound microscope (Primostar, Carl Zeiss, Oberkochen, Germany).

### 2.3. Molecular Identification of Fungal Isolates by Using Internal Transcribed Region and Tef-1α Gene

#### 2.3.1. DNA Extraction

Based on morphological characteristics, ten *Fusarium* spp. isolates were grown in potato dextrose broth for mycelium production to be used for DNA extraction. DNA was extracted from mycelium harvested from broth media based on the cetyl trimethyl ammonium bromide (CTAB) method [34] with a slight modification. About 0.2 g of mycelium of each isolate was ground separately to a fine frozen powder in a sterile pre-chilled mortar and pestle using liquid nitrogen, after which a DNA extraction buffer (100 mM Tris HCl at pH 8.0, 1.4 M NaCl, 50 mM EDTA at pH 8.0, and 2% CTAB) was added. The RNA present with DNA was removed by RNase treatment. Quantification of DNA was performed by nano spectrophotometer (MicroDigital, Seoul, Republic of Korea). Working solutions of 25 ng/µL were prepared for optimization of polymerase chain reaction (PCR) by adding double-sterilized nuclease-free water.

#### 2.3.2. Polymerase Chain Reaction

The internal transcribed spacer (ITS1/4) and translation elongation factor (Tef) alpha fragments were amplified using the primer pairs (5′-TCCGTAGGTGAACCTGCGG-3′ and 5′-TCCTCCGCTTATTGATATGC-3′) and (5′-CATCGAGAAGTTCGAGAAGG-3′ and 5′-TACTTGAAGGAACCCTTACC-3′), respectively. The PCR reactions were carried out in a 20 μL reaction mixture containing 10× PCR buffer, 50 ng DNA template, 1.5 mM MgCl_2_, 0.25 mM dNTP mixture, 0.25 μM each of primer, and one unit of Dream Taq Polymerase (Thermo Scientific, Mumbai, India). PCR reactions were run in Applied Biosystems Thermocycler, Foster City, CA, USA, with the following settings: denaturation at 94 °C (5 min); then 35 cycles of 94 °C (30 s), 56 °C (45 s), and 72 °C (90 s). The final extension was performed at 72 °C for 10 min.

#### 2.3.3. Sequencing and Phylogenetic Analysis

The ITS and *Tef*-1α sequences obtained were BLAST-searched against the NCBI GenBank database to confirm the identity of the strains. A phylogenetic tree was established from concatenated tef gene sequences using the Maximum Likelihood method in the molecular evolutionary genetics analysis tool MEGA ver. 11 [35].

#### 2.3.4. Pathogenicity Assay

The *Fusarium* spp. isolated from the host plants were cultured and tested for their pathogenicity to the onion cultivar Bhima Super. The seedling pathogenicity assay and the onion bulb inoculation assay was performed for ten isolates of *Fusarium* spp. (OGRDWF1 to OGRDFW10). Fifty-day-old seedlings were used for the pathogenicity assay and the experiment was conducted in controlled conditions under a net house. The experimental design was completely randomized and constituted in three replications. The seedling pathogenicity assay was carried out by dipping roots in the conidial suspension of ten *Fusarium* spp. isolates independently (10^6^ conidia/mL) for 15 min and then transplanting them into pots containing sterilized potting mix [13,24]. Each seedling was then drenched at the base with 5 mL of conidial suspension (10^6^ conidia/mL). The control plants were treated with only sterile distilled water. According to Kalman et al. [36], the bulb pathogenicity assay was carried out by injecting a conidial suspension (10^6^ conidia/mL) of the ten *Fusarium* spp. isolates independently into the basal plate of the bulbs (n = 40) and incubating the bulbs in moisture bags in the dark at 25 ± 2 °C for one week. After one week of incubation, external and internal disease symptoms were observed until 20 days and evaluated qualitatively, and the fungus from infected seedlings and bulbs was re-isolated and cultured to satisfy Koch’s postulates.

## 3. Results

### 3.1. FBR Incidence in Maharashtra

**Figure 1:**
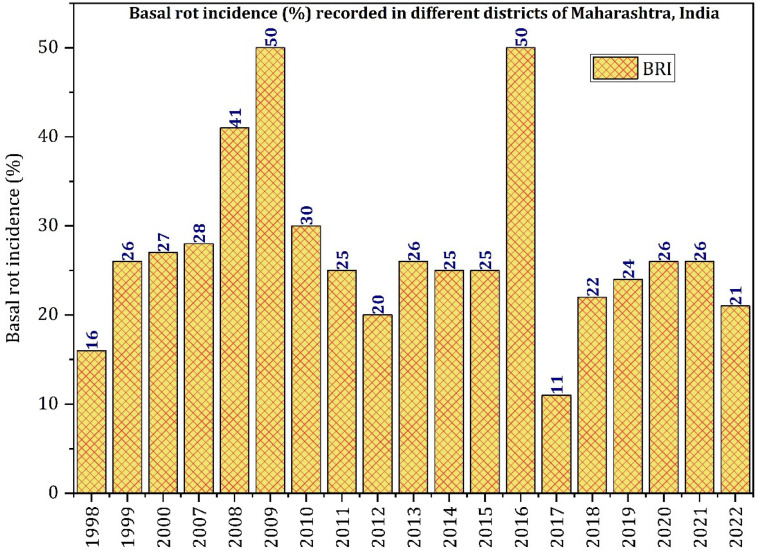
Disease incidence of *Fusarium* basal rot disease in the Maharashtra state of India from 1998 to 2022.

**Figure 2:**
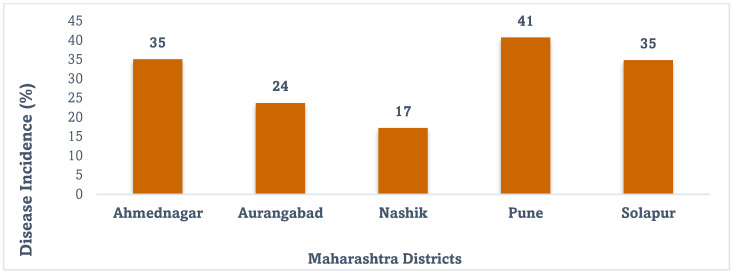
Disease incidence of *Fusarium* basal rot disease in the Maharashtra state of India during 2023.

The analysis of reports of data from disease surveys of onion-growing areas of Maharashtra from 1998 to 2022 was carried out from available reports of ICAR-DOGR (1998–2020) and AINROPG (2021–2022) with missing years from 2001 to 2006, where the *Fusarium* basal rot (FBR) incidence varied from 11 to 50% (Figure 1). The disease incidence recorded during the fresh survey in 2023 of five different districts of Maharashtra, viz., Ahmednagar, Aurangabad, Nashik, Pune, and Solapur, varied from 17 to 41% (Figure 2).

**Figure 3:**
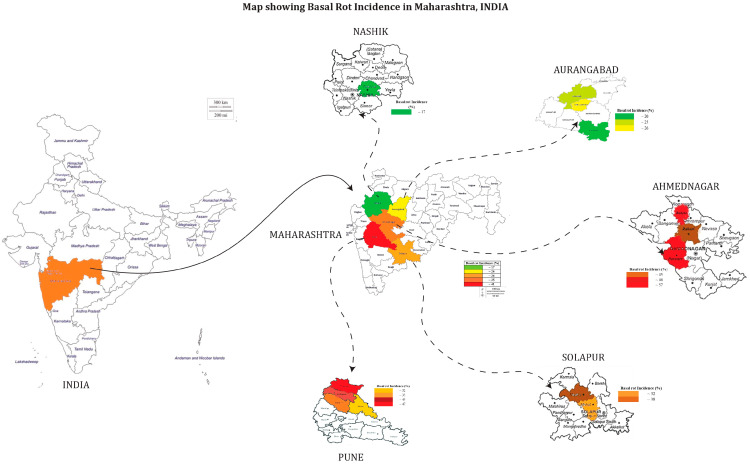
Map showing the onion basal rot incidence in Maharashtra.

In our survey during 2023, we noticed that the highest disease incidence of 80.50% was observed in the village of Tejewadi in Ambegaon block followed by the village of Belhe in Junnar block of Pune district with an incidence of 78.50%. The lowest basal rot incidence (12.00%) was observed in the village of Niphad in Nashik district (Table 1; Figure 3). The highest average FBR incidence (40.64%) was recorded in Pune district followed by Ahmednagar (35.00%), Solapur (34.75%), and Aurangabad (23.67%), and the lowest average incidence of 17.17% was recorded in Nashik district (Figure 2). The native isolates of *Fusarium* spp. were isolated from respective locations and designated as OGRDFW1 to OGRDFW10.

### 3.2. Isolation and Identification of Pathogens Causing Basal Rot

**Figure 4:**
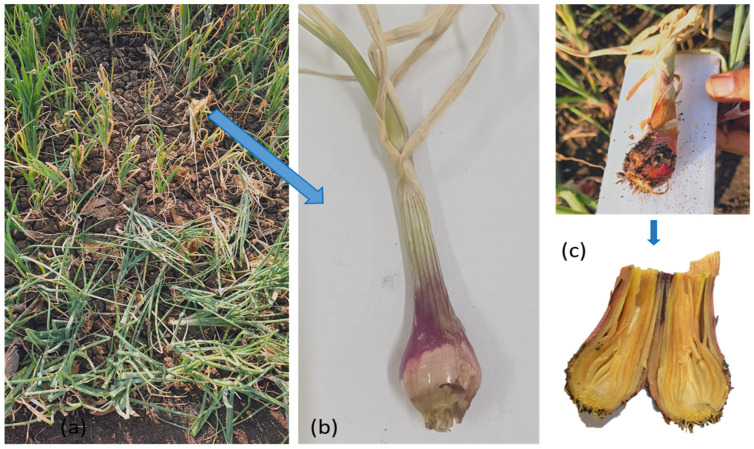
Varying symptoms of *Fusarium* Basal rot of onion: (**a**) FBR-infected field. (**b**) Single plant from the field with leaf dieback, rotting of the basal plate, and loss of roots. (**c**) Intact infected plant and split open bulb showing soft watery decay.

The disease symptoms observed on onion plants and bulb rot spreading from the onion basal plate upwards in the scales were selected and subjected to isolation and morphological identification (Figure 4). The isolates were purified and kept for incubation at 25 ± 2 °C. After 7 days of incubation, the isolates were preliminarily identified based on morphological characteristics. The radial growth of all the isolates varied from 64 to 80 mm mycelial growth at 7 days of incubation.

**Figure 5:**
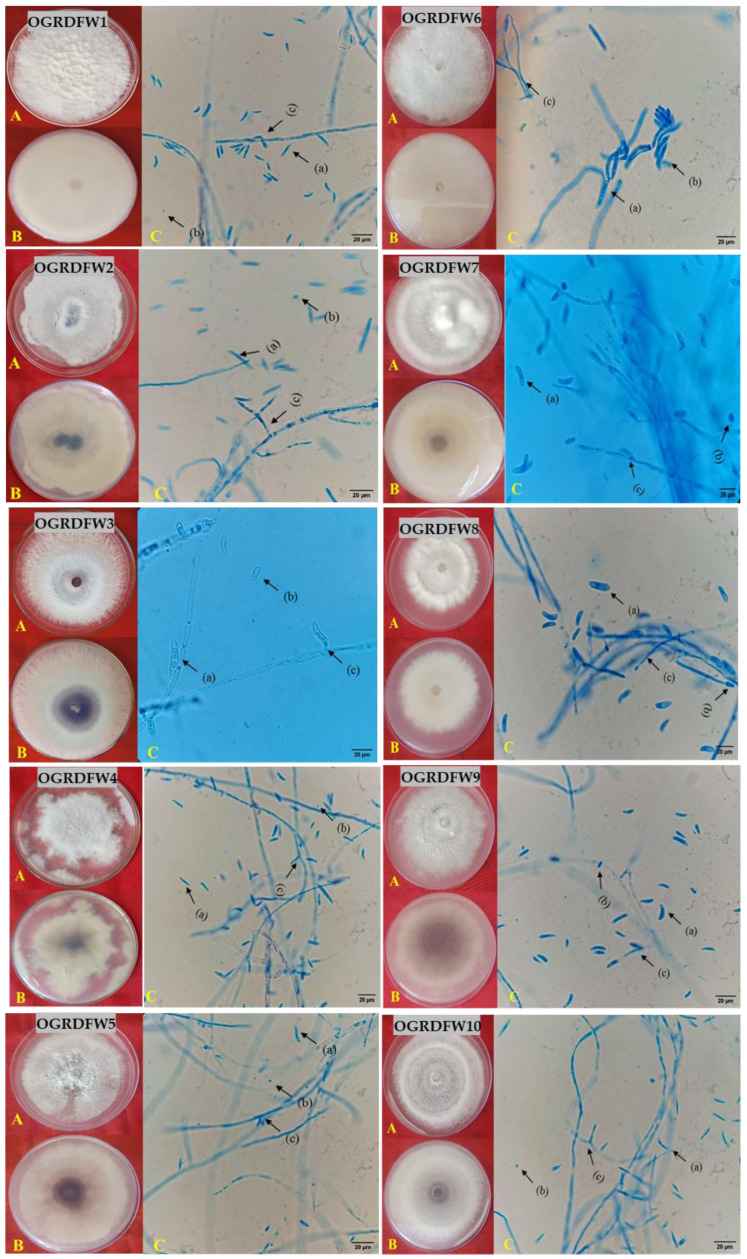
Colony morphology and microscopic characterization of the *Fusarium* spp. isolates: (**A**) top view of mycelia, (**B**) bottom view, (**C**) microscopic field (1000×), (a) macroconidia, (b) microconidia, and (c) spore-bearing structure (sporophyte).

The colonies of *F. acutatum* isolates showed white aerial mycelium that was purplish to pinkish in the center on both sides of Petri plates while the *F. falciforme* isolates showed white aerial mycelium on both sides (Table 2, Figure 5). When the cultures grew old, they became thick due to the growth of white aerial hyphal mats on the colony surface. Each isolate was observed with macroconidia and microconidia and their size was measured.

The macroconidia of *F. acutatum* were hyaline and falcate with bent apical cells with 1–2 septa measuring 18.2 to 20.3 × 2.4 to 3.7 μm (n = 40), while microconidia were ovoid and aseptate, measuring 3.6 to 5.8 × 3.2 to 4.3 μm (n = 40). The chlamydospores were round intercalary, hyaline, and single (Table 2, Figure 5). Macroconidia of *F. falciforme* were a little bigger measuring 25.2 to 30.3 × 4.0 to 5.3 μm (n = 40), hyaline in color and falcate with slightly curved apexes with 2–3 septa, while the microconidia were 9.8 to 12.4 × 2.1 to 2.7 μm (n = 40), cylindrical to ellipsoid in shape, hyaline in color, and with 0–1 septa. The chlamydospores were round and intercalary, either single or in chains (Table 2, Figure 5).

### 3.3. Molecular Identification of Fusarium spp. Isolates Using Internal Transcribed Region (ITS) and Translation Elongation Factor (Tef)1α Gene

The isolated pathogens were morphologically identified as *Fusarium* spp. and further subjected to molecular characterization using universal ITS and *tef* gene primers for confirmation. The sequence analysis of *F. acutatum* and *F. falciforme* with ITS 1/4 resulted in amplicons of 338 to 365 and 357 bp, while the amplicon sizes of the tef gene were 524 to 652 and 652 to 656 bp, respectively. The BLAST tool of NCBI (National Centre for Biotechnology Information) identified the isolates OGRDFW1, OGRDFW2, OGRDFW4, OGRDFW5, OGRDFW6, and OGRDFW10 as *F. acutatum* (>99% similarity), while the isolates OGRDFW3, OGRDFW7, OGRDFW8, and OGRDFW9 showed similarity with *F. falciforme* (Table 3). After identification, the three representative isolates from *F. acutatum* (NAIMCC-F-04492, NAIMCC-F-04493, and NAIMCC-F-04494) and one from *F. falciforme* (NAIMCC-F-04495) were deposited in the National Agriculturally Important Microbial Culture Collection (NAIMCC), Mau, Uttar Pradesh (India), and assigned accession numbers.

### 3.4. Phylogenetic Relationship Based on ITS rRNA and Tef-1α Gene Sequences

**Figure 6:**
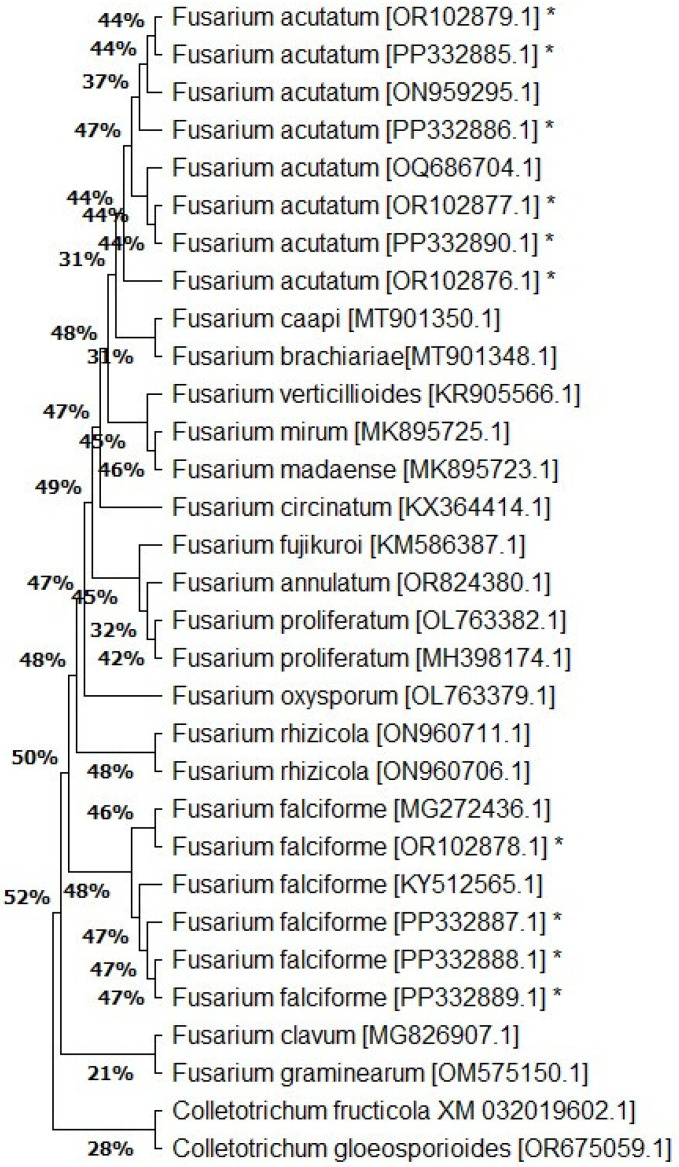
Phylogenetic tree showing the relationship of 10 *Fusarium* isolates based on tef1-α gene sequence using the Maximum Likelihood method. The percentage of replicate trees in which the linked taxa clustered together in the bootstrap test (1000 replicates). *Colletotrichum fructicola* and *Colletotrichum gloeosporioides* were used as an out-group (* isolates included in this study).

Evolutionary history was inferred by using the Maximum Likelihood method and Tamura–Nei model. Initial tree(s) for the heuristic search were obtained automatically by applying Nearest Neighbor Interchange (NNI) algorithms to a matrix of pairwise distances estimated using the Tamura–Nei model, and then selecting the topology with the superior log likelihood value. The tree was drawn to scale, with branch lengths measured in the number of substitutions per site. This analysis involved 31 nucleotide sequences. Evolutionary analyses were conducted in MEGA11. The phylogenetic analysis based on *Tef*-1α sequences revealed that six isolates (OGRDFW1, OGRDFW2, OGRDFW4, OGRDFW5, OGRDFW6, and OGRDFW10) belong to *F. acutatum*, whereas four isolates (OGRDFW3, OGRDFW7, OGRDFW8, and OGRDFW9) belong to *F. falciforme*. Nodal robustness was targeted by the bootstrap method. The numerical value presented in the node indicates the bootstrap value. Based on the bootstrap values, isolates were clustered with *F. acutatum* and *F. falciforme*. The ITS sequences of four isolates and *tef1* gene sequences of all ten isolates were submitted to GenBank (Table 3, Figure 6).

### 3.5. Pathogenicity Assay

The pathogenicity of six *F. acutatum* and four *F. falciforme* isolates was tested on seedlings and bulbs of a susceptible variety Bhima Super. All inoculated seedlings received infection and developed typical visible symptoms of FBR, viz., yellowing and wilting, and were all dead by 20 dpi (days post inoculation). And the inoculated bulbs also developed the soft rotting symptoms of FBR by 15 dpi and developed tissue decay of bulbs accompanied by the outgrowth of white hyphae on the bulb surface exhibited at 20 dpi, while the un-inoculated seedlings and bulbs remained healthy. The symptoms that developed in this inoculation assay were similar to those seen in naturally infected onion fields both on seedlings and bulbs. The pathogens *F. falciforme* and *F. acutatum* were reisolated as the original isolate, thus fulfilling Koch’s postulates.

## 4. Discussion

*Fusarium* basal rot (FBR) is an economically important disease, causing major loss in both pre-harvest and post-harvest conditions. Since the disease was reported, *F. oxysporum* f. sp. *cepae* has been known to be the causal agent of basal rot of onion [21,37,38,39] in India. Shamyuktha et al. [39] recorded 27.00 to 74.33% *F. oxysporum* f. sp. *cepae* incidence in the Tirunelveli, Thoothukudi, and Tenkasi districts of Tamil Nadu. Muthukumar et al. [40] also reported a basal rot incidence varying from 10.12 to 24.87%, caused by *F. oxysporum* f. sp. *cepae*, in all surveyed locations of Tamil Nadu. Even recently, Thakare et al. [30] carried out a survey mostly in the locations covered in this study, in the Nashik, Pune, Solapur, and Ahmednagar districts of Western Maharashtra, and recorded the basal rot incidence caused by *F. oxysporum* f. sp. *cepae* with a range of 27.09 to 38.83%. In their survey, the Nashik district recorded the highest incidence, while Solapur recorded the lowest, whereas in our survey the Nashik district recorded the lowest disease incidence, and we did not record *F. oxysporum* f. sp. *cepae* in our collected samples.

Ravi et al. [22] observed the FBR incidence on onion bulbs in major growing areas of the Kadapa and Kurnool districts of Andhra Pradesh and reported *F. proliferatum* as the causal agent of FBR. Recently, *F. falciforme* [13] and *F. acutatum* [24] were also reported in Karnataka as causal agents of FBR.

In Maharashtra, basal rot, aka FBR, was reported to be caused by *Fusarium oxysporum* f. sp. *cepae* until 2023 [28,29,30,31]. To understand the association and active prevalence of different *Fusarium* spp. causing basal rot in Maharashtra, a survey was conducted in five districts, viz., Ahmednagar, Aurangabad, Nashik, Pune, and Solapur of Maharashtra, in 2023 and the affected samples were collected and subjected to isolation and identification. Survey results revealed the presence of FBR incidence in surveyed locations of Maharashtra. The average incidence of FBR was the highest in Pune and the lowest in the Nashik district. After analyzing the basal rot incidence and the samples collected, it was revealed that, of the ten samples isolated and verified, four were caused by *F. falciforme* and six by *F. acutatum*. Our results are in agreement with Sarwadnya et al. [13], who also reported *F. falciforme* causing FBR in Karnataka. Bhat et al. [24] conducted a survey in the Bangalore, Bagalkot, Chitradurga, and Hassan districts of Karnataka and reported that FBR was caused by *F. acutatum* and *F. falciforme*.

The ICAR-DOGR (the then NRCOG), Pune, Maharashtra, initiated sporadic surveys from 1998 to record the status of basal rot from Maharashtra, and the analysis of the disease data revealed a variable intensity ranging from 11 to 50% [25,26,27]. After going through the recent updates of reports from Karnataka to Maharashtra, the association of *F. acutatum* and *F. falciforme* in the previous year too could not be ruled out.

The plant and bulb samples collected from each of the 38 locations in the Ahmednagar, Aurangabad, Nashik, Pune, and Solapur districts were subjected to isolation. Pathogen diagnosis was carried out as described by Sarwadnya et al. [13] and Bhat et al. [24]. Single hyphal tips were plated on potato dextrose agar and pure cultures were obtained, which were used for characterization.

The morphological characteristics of *Fusarium* spp. have been used in the past as the preferred method for species identification. The colonies of *F. acutatum* isolates had a white aerial mycelium with a reddish to pinkish center, but the isolates of *F. falciforme* displayed a white aerial mycelium on both sides. All these observations were found to be similar to those reported by Sarwadnya et al. [13] and Bhat et al. [24]. Earlier, Kalman et al. [36] studied the morphology of *Fusarium acutatum* and observed fungal colonies of white aerial mycelium and growth rates ranging from 1.07 to 1.24 cm/day, and the size of the microconidia was 5.8–9.75 × 2.4–2.7 µm and macroconidia varied from 18.2 to 57.7 × 3.1 to 3.7 µm.

Since the *Fusarium* genus is complex, and morphological differences make it challenging to differentiate species [6,41], the isolates of the *Fusarium* spp. obtained from infected plants and bulbs of onion plants could not be differentiated morphologically; therefore, for further confirmation of identification, the *Fusarium* spp. isolates were subjected to molecular characterization using ITS1/4 and *tef* gene primers [13]. The sequences were BLAST-searched in the NCBI database, confirming the identity of *Fusarium* spp. [42]. The Tef1-α sequences were submitted to GenBank, which are available with the accession numbers of *F. acutatum* (OR102876, OR102877, OR102879, PP332885, PP332886, PP332890) and *F. falciforme* (OR102878, PP332887, PP332888, PP332889), respectively. The phylogenetic analysis clearly differentiated the species. The tef gene nucleotide sequences revealed that *F. acutatum* and *F. falciforme* clustered in a distinct phylogenetic clade from other species of *Fusarium*. Our findings are similar to those described by Bhat et al. [24] based on the multi-locus BLAST analysis in the Fusarioid-ID database and showed that 14 isolates belonged to *F. falciforme* [13] and 12 belonged to *F. acutatum*.

In the pathogenicity assay, six *F. acutatum* and four *F. falciforme* were used on seedlings and bulbs of a susceptible ‘Bhima Super’ variety. The identity of the two species (*F. acutatum and F. falciforme*) as the causal agents of onion bulb and seedling rot disease was predominantly approved here using Koch’s postulates [13]. At 20 dpi, typical symptoms observed were yellowing, wilting, and death of seedling in bulbs; tissue decay with white mycelial growth on basal plates; and uninoculated plants remained healthy. Some earlier works studied the pathogenicity of *Fusarium oxysporum* f. sp. *cepae* on onion and observed the typical symptoms of yellowing and eventual dieback of the leaves, the drying of leaves, and, in the advanced stage, complete rotting of the basal portion of plants and the death of plants at 15 dpi [15,21,38,43,44].

The reports from Iran, Finland, and Burkina Faso suggest that *F. solani* and *F. redolens* typically result in lesions on seedlings, while they occur as asymptomatic infections on bulbs [10,45,46]. Seedling age and growth stage have been proposed as major determinants of FBR presence on *Allium* spp. FBR tends to be more aggressive when infection establishes at an early stage of seedling development and on mature bulbs in storage [6,9,47,48]. Le et al. [23] studied the co-infection of *F. solani, F. proliferatum*, and *F. oxysporum* on onion, where *F. solani* was highly virulent on seedlings while *F. proliferatum* and *F. oxysporum* severely affected both bulbs and seedlings in Vietnam.

As evident from the above reports, the causal agent for basal rot in Maharashtra was thought to be only caused by *F. oxysporum* f. sp. *cepae* until 2023 [30], and we have now found both *F. falciforme* and *F. acutatum* to be associated with FBR, which is the first report from Maharashtra and indicates the active prevalence of *F. falciforme* and *F. acutatum*.

## 5. Conclusions

There is a high probability that *F. falciforme* and *F. acutatum* contribute significantly to basal rot disease and were not reported earlier in Maharashtra. Although *F. falciforme* and *F. acutatum* have been previously reported in India, and the prevalence of the disease is now actively increasing in India. Though earlier report says that *F. oxysporum* f. sp. *cepae* is a major pathogen in causing FBR in Maharashtra. Our survey results showed that the *F. falciforme* and *F. acutatum* damage in onions ranged from 17 to 41%. Thus, the active prevalence of FBR was confirmed in Maharashtra, and to the best of our knowledge, this is the first report of *F. falciforme* and *F. acutatum* causing basal rot of onion independently in Maharashtra, India.

## Figures and Tables

**Table 1:** Survey locations in 2023 with their coordinates and FBR incidences.

Sl. No.	District	Block	Village	Latitude	Longitude	FBR Incidence (%)
1	Ahmednagar	Parnner	Chombut	19.02666	74.20852	35.00
2	Ahmednagar	Parnner	Chombut	19.02568	74.20462	46.00
3	Ahmednagar	Parnner	Pimpalner	20.91374	74.12899	48.00
4	Ahmednagar	Parner	Alkuti	19.05379	74.22518	56.00
5	Ahmednagar	Rahuri	Aaradgaon	19.42520	74.70284	42.00
6	Ahmednagar	Rahuri	Sade	19.34345	74.67564	44.00
7	Ahmednagar	Rahuri	Digras	20.09561	77.73518	49.50
8	Ahmednagar	Rahata	Sakori	19.34345	74.47938	56.50
					Mean	**35.00**
9	Aurangabad	Paithan	Dongaon	20.21854	75.16469	20.00
10	Aurangabad	Kannad	Bahirgaon	20.22815	75.16196	25.00
11	Aurangabad	Khulatabad	Talyachiwadi	18.85495	77.16845	26.00
					Mean	**23.67**
12	Nashik	Niphad	Chitegaon	20.00766	73.95402	20.00
13	Nashik	Niphad	Chitegaon	20.00641	73.95663	19.50
14	Nashik	Niphad	Niphad	20.00875	73.94513	12.00
					Mean	**17.17**
15	Pune	Ambegaon	Kathapur Kh	18.99281	74.11083	40.00
16	Pune	Ambegaon	Pimparkhed	18.99313	74.13356	39.50
17	Pune	Ambegaon	Ranjani	19.03569	74.05341	32.00
18	Pune	Ambegaon	Tejewadi	19.19511	73.94248	80.50
19	Pune	Ambegaon	Thorandale	19.03197	74.02799	34.00
20	Pune	Junnar	Amrapur	19.19611	73.90603	69.50
21	Pune	Junnar	Belhe	19.10992	74.15500	78.50
22	Pune	Junnar	Pimpalgaon Tarfe Narayangaon	19.15909	73.91505	21.00
23	Pune	Junnar	Wadgaon Sahani	19.18725	74.18502	34.50
24	Pune	Junnar	Pimpali Pendhar	19.21887	74.04609	46.00
25	Pune	Junnar	Pipmari Kawal	19.01865	74.17225	30.00
26	Pune	Khed	Chandoli Bk	18.83690	73.87903	60.50
27	Pune	Khed	Khalachi Bhamburwadi	18.85087	73.89857	14.00
28	Pune	Khed	Chinchbaiwadi	18.87634	73.97794	49.50
29	Pune	Khed	Pabal	18.82956	74.04781	58.50
30	Pune	Khed	DOGR	18.50358	73.53050	21.00
31	Pune	Khed	Manjri	18.52264	73.97608	14.50
32	Pune	Khed	Manchar	19.00552	73.94635	26.00
33	Pune	Shirur	Chandoh	18.99871	74.16710	28.20
34	Pune	Shirur	Jambut	19.00590	74.18010	35.00
					Mean	**40.64**
35	Solapur	Mohol	Yawali	17.84161	75.59669	26.00
36	Solapur	Mohol	Lamboti	17.78573	75.71151	38.00
37	Solapur	Madha	Bhosare	17.60582	74.33768	35.00
38	Solapur	Madha	Tembhurni	18.02982	75.18970	40.00
					Mean	**34.75**

**Table 2:** Morphological characterization and microscopic identification of *Fusarium* spp. isolates.

Isolate No.	*Fusarium* spp.	Radial Growth at 7 Days (mm)	Colony Morphology	Macroconidia (µm)	Microconidia (µm)
OGRDFW1	*F. acutatum*	80	White aerial rough mycelium, irregular margin, reverse side whitish	18.2 × 3.7	4.9 × 2.1
OGRDFW2	*F. acutatum*	77	White aerial mycelium with dark purplish center, flat, irregular margin, reverse side white to purple with concentric rings	18.3 × 2.4	4.6 × 2.1
OGRDFW3	*F. falciforme*	78	White raised aerial mycelium, regular margin, reverse side whitish	30.3 × 5.3	12.4 × 3.3
OGRDFW4	*F. acutatum*	65	White aerial mycelium with purplish to pinkish center, flat, regular margin, reverse side purple to pinkish with concentric rings	18.2 × 3.7	5.8 × 2.7
OGRDFW5	*F. acutatum*	78	White mycelium with purplish center, flat, regular margin, reverse side white to purple with concentric rings	20.3 × 3.2	4.2 × 2.0
OGRDFW6	*F. acutatum*	80	White aerial mycelium, regular margin, reverse side whitish	18.6 × 3.5	4.5 × 2.6
OGRDFW7	*F. falciforme*	80	White aerial fluffy mycelium, irregular margin, reverse side white with faint yellowish center	25.2 × 5.0	10.0 × 4.3
OGRDFW8	*F. falciforme*	64	White raised aerial mycelium, irregular margin, crateriform, reverse side creamish	28.4 × 4.8	11.2 × 3.9
OGRDFW9	*F. falciforme*	76	Purple-white aerial mycelium, flat, regular margin, reverse side purple to pinkish with concentric rings	26.8 × 4.0	9.8 × 3.2
OGRDFW10	*F. acutatum*	77	White with purple aerial mycelium with concentric rings, flat, regular margin, reverse side white to purple with concentric rings	19.5 × 2.9	3.6 × 2.1

**Table 3:** BLASTN identification of the *Fusarium* spp. isolates from this study.

*Fusarium* spp.	Isolate	Gene	NCBI Accession	Amplicons (bp)	NCBI Identity (%)	Collection Site
*F. acutatum*	OGRDFW1	ITS	OR084795	338	99.51	Khed, Pune
		TEF1	OR102876	613		
*F. acutatum*	OGRDFW2	ITS	OR084796	352	99.67	Khed, Pune
		TEF1	OR102877	613		
*F. falciforme*	OGRDFW3	ITS	OR084797	357	99.23	Khed, Pune
		TEF1	OR102878	652		
*F. acutatum*	OGRDFW4	ITS	OR084798	365	99.36	Khed, Pune
		TEF1	OR102879	627		
*F. acutatum*	OGRDFW5	TEF1	PP332885	614	98.80	Niphad, Nashik
*F. acutatum*	OGRDFW6	TEF1	PP332886	524	99.70	Ambegaon, Pune
*F. falciforme*	OGRDFW7	TEF1	PP332887	658	95.14	Rahuri, Ahemdanagar
*F. falciforme*	OGRDFW8	TEF1	PP332888	658	96.12	Paithan, Aurangabad
*F. falciforme*	OGRDFW9	TEF1	PP332889	656	95.08	Mohol, Solapur
*F. acutatum*	OGRDFW10	TEF1	PP332890	614	99.69	Madha, Solapur

## Data Availability

Data are contained within the article.
